# 卡拉西珠单抗联合血浆置换、糖皮质激素及利妥昔单抗治疗难治性血栓性血小板减少性紫癜1例报告并文献复习

**DOI:** 10.3760/cma.j.cn121090-20251126-00553

**Published:** 2026-03

**Authors:** 求哲 魏, 聪 卢, 慧雯 江, 俊斌 胡, 耀辉 吴, 豫 胡

**Affiliations:** 华中科技大学同济医学院附属协和医院血液内科，武汉 430022 Department of Hematology, Union Hospital, Tongji Medical College, Huazhong University of Science and Technology, Wuhan 430022, China

## Abstract

回顾性分析华中科技大学同济医学院附属协和医院血液科收治的1例难治性免疫性血栓性血小板减少性紫癜（iTTP）患者的诊疗经过，并结合相关文献进行复习。该患者入院时PLASMIC评分为6分，立即启动治疗性血浆置换（TPE）联合糖皮质激素治疗。进一步检查示其血管性血友病因子裂解蛋白酶（ADAMTS13）活性为2.9％且抑制物阳性，符合iTTP诊断。经连续5 d的TPE联合糖皮质激素治疗后，患者无明显临床反应，符合难治性iTTP诊断；随后加用利妥昔单抗，但血小板仍持续下降且病情进一步加重。在原方案基础上联合使用卡拉西珠单抗后，患者的血小板与乳酸脱氢酶迅速恢复至正常水平，ADAMTS13活性回升至24.9％，且治疗期间未发生严重不良事件。本病例提示，对于常规治疗无效的难治性iTTP患者，将卡拉西珠单抗纳入联合治疗策略能够有效实现疾病的快速控制。

## Abstract (en)

The clinical course and management of a patient with refractory immune thrombotic thrombocytopenic purpura（iTTP）admitted to the Department of Hematology, Union Hospital, Tongji Medical College, Huazhong University of Science and Technology, were retrospectively analyzed, alongside a review of pertinent literature. Upon admission, the patient had a PLASMIC score of 6, and emergency therapeutic plasma exchange（TPE）combined with glucocorticoid therapy was immediately initiated. Further examinations revealed an ADAMTS13 activity of 2.9％ and a positive inhibitor, thereby confirming iTTP diagnosis. After 5 consecutive days of TPE and glucocorticoid treatment, the patient demonstrated no clinical response, consistent with a diagnosis of refractory iTTP. Rituximab was subsequently added; however, the platelet count continued to decrease, and the disease progressed. After adding caplacizumab to the original regimen, the platelet and lactate dehydrogenase levels rapidly normalized, ADAMTS13 activity recovered to 24.9％, and no serious adverse events occurred during the treatment. This case indicates that incorporating caplacizumab into the combination therapy strategy for patients with refractory iTTP who failed conventional therapies effectively achieves rapid disease control.

血栓性血小板减少性紫癜（thrombotic thrombocytopenic purpura，TTP）是一种危及生命的血栓性微血管病，它是由血小板过度聚集和微血管血栓导致微血管性溶血性贫血（microangiopathic hemolytic anemia，MAHA）、血小板减少以及器官功能障碍所引起的全身性疾病[1]。其病因是血管性血友病因子（von Willebrand factor，vWF）裂解蛋白酶（ADAMTS13）严重缺乏，当ADAMTS13活性严重降低（<10％）时，大量vWF多聚体会积聚并与血小板结合，形成广泛微血管血栓，从而导致器官缺血性损伤（例如神经系统症状、肾功能损害等）[2]。根据ADAMTS13缺乏机制不同，TTP主要分为遗传性TTP（cTTP，常染色体隐性遗传，由ADAMTS13基因的双等位基因突变引起，患者是纯合子或者复合杂合子）和免疫性TTP（iTTP，由针对ADAMTS13的自身抗体所引起，占TTP 95％以上）[2]–[3]。目前TTP首选治疗性血浆置换（therapeutic plasma exchange，TPE），并酌情联合使用糖皮质激素、利妥昔单抗等[4]。卡拉西珠单抗（caplacizumab）是一种抗vWF的人源化双价、仅含可变结构域的免疫球蛋白片段，可高亲和力、特异性地阻断超大分子量vWF多聚体与血小板GPⅠb-Ⅸ-Ⅴ受体的相互作用，从而从上游抑制血小板黏附和微血栓的形成[5]。其临床意义在于能够快速阻断TTP的核心病理环节，显著缩短血小板恢复时间和减少急性期弥漫性器官损伤及死亡的风险[2],[6]。目前国内关于TTP患者接受卡拉西珠单抗治疗的数据仍然有限。本研究报道1例接受卡拉西珠单抗治疗的iTTP患者的病例资料，并进行文献复习。

## 病例资料

患者，女，36岁，因“乏力1周余，发现血小板减少2 d”于2025年10月6日入我科普通病房住院治疗。患者入院前1周无明显诱因出现乏力、头晕，四肢出现瘀斑，10月4日当地医院查血常规示：PLT 20×10^9^/L、HGB 91 g/L，输注血小板1次；10月5日出现发热，体温最高37.6°C，无鼻塞流涕，无咳嗽咳痰；10月6日凌晨出现右上肢麻木，伴吐词欠清，急查头部CT未见明显异常。手术史：2025年4月因甲状腺乳头状癌行右侧甲状腺切除术；婚育史：已婚，妊娠2次产1次。入院查体：神清，精神差，生命体征平稳，皮肤巩膜无黄染，全身皮肤黏膜多发出血点、紫癜和瘀斑，肝脾肋缘下未触及，双下肢无凹陷性水肿。PLASMIC评分（0～4分为低危，5分为中危，6～7分为高危）为6分（**扫描本文首页二维码查阅附表1**），TTP可能性极大。入院后立即启动地塞米松15 mg/d及TPE（1 500 ml新鲜冰冻血浆联合5％白蛋白，每日1次）治疗。

入院后实验室检查：①血常规：PLT 12×10^9^/L，HGB 86 g/L；②肝肾功能：总胆红素72.3 µmol/L，间接胆红素59.4 µmol/L，丙氨酸氨基转移酶45 U/L，天冬氨酸氨基转移酶26 U/L，乳酸脱氢酶（LDH）1 199 U/L，肌酐65.3 µmol/L，尿素氮4.92 mmol/L；③ADAMTS13活性2.9％，ADAMTS13抑制性抗体检测阳性；④妊娠试验阴性；⑤感染相关：病毒性肝炎系列、人类免疫缺陷病毒及梅毒螺旋体抗体筛查均阴性，呼吸道病原体及常见嗜血病毒相关抗体未见异常；⑥自身免疫相关检查：抗核抗体滴度1∶100，抗磷脂抗体、狼疮抗凝物质、类风湿因子等均呈阴性；⑦凝血功能基本正常，肌钙蛋白I（TnI）89.3 ng/L。患者10月9日PLT升至46×10^9^/L。骨髓穿刺未提示其他血液系统肿瘤，外周血破碎红细胞5.4％。综合以上实验室检查结果并排除其他血栓性微血管病及血液系统肿瘤后，最终确诊为iTTP。10月10日加用利妥昔单抗375 mg/m^2^，每周1次；10月11日将地塞米松15 mg/d换为甲泼尼龙280 mg/d；10月12日夜间患者出现神志障碍，呼之不应，不自主左右摇头，查体不配合，紧急行头颅CT检查未见明显出血、梗死，患者神志障碍进一步加重，出现双眼向上凝视，双侧瞳孔较前缩小，肌张力增高，予以地西泮对症处理后转入血液科重症监护病房（HCU），转入时急性生理与慢性健康状况评分系统Ⅱ（APACHEⅡ）评分23分，序贯器官衰竭评分（SOFA评分）11分。急查血常规示：PLT 9×10^9^/L，HGB 69 g/L，LDH较前明显升高，患者已行TPE及激素治疗7 d，尚未取得临床反应甚至病情明显加重，符合难治性iTTP诊断。转入HCU后调整治疗方案：①控制原发病：继续每日1次TPE，甲泼尼龙暂维持原剂量。②卡拉西珠单抗：由于当时该药尚未在国内全面上市，本院在遵循临床急需原则下，经医院医务处审核备案，由主管医师向患者及家属充分告知其获益与潜在风险，家属表示理解并签署了知情同意书，购药及使用全程符合医疗伦理规范。治疗第1天（10月16日）在TPE前至少15 min静脉注射11 mg，TPE后皮下注射11 mg；治疗第2、3天TPE后皮下注射11 mg。③支持治疗：抗癫痫、抗感染、营养支持。10月18日复查PLT 121×10^9^/L，LDH水平降至209 U/L；10月19日PLT恢复至164×10^9^/L，LDH 193 U/L，停止TPE，继续甲泼尼龙治疗；10月21日复查PLT 191×10^9^/L，ADAMTS13活性24.9％，ADAMTS13抑制物0.1 BU，外周血破碎红细胞2.4％，APACHEⅡ评分16分，SOFA评分5分，提示当前病情已由危重期过渡至相对稳定阶段。患者脱离血浆置换后PLT基本稳定，自主意识恢复，抗癫痫药物逐渐减停，症状明显好转；尤其卡拉西珠单抗给药后，患者格拉斯哥昏迷评分（Glasgow coma scale，GCS）迅速由9分恢复至14分，神经系统症状明显改善。患者治疗过程中重要指标变化见图1，神经系统症状及GCS变化见附表2（**扫描本文首页二维码查阅**）。

**图1:**
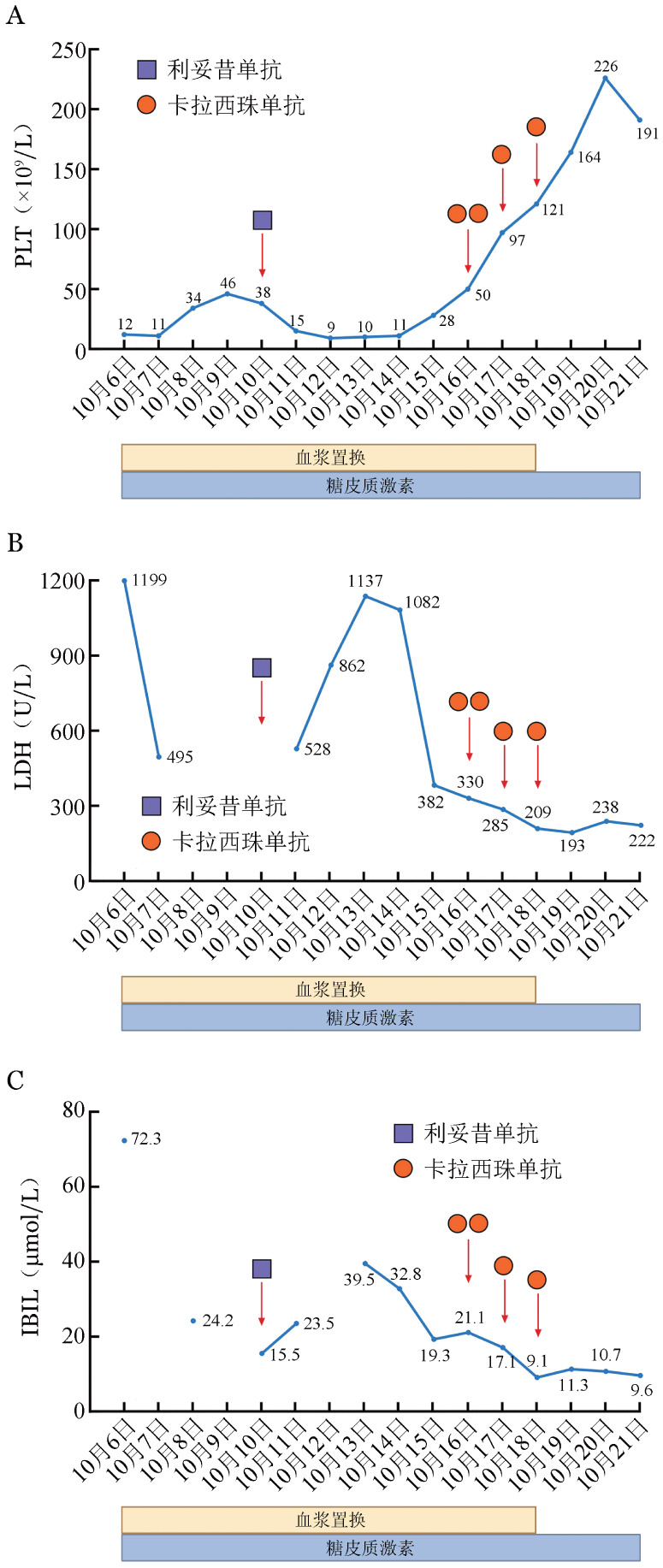
血栓性血小板减少性紫癜（TTP）患者治疗过程中重要指标变化 **A** 血小板计数（PLT），患者入院后立即启动治疗性血浆置换及糖皮质激素治疗，PLT短暂回升后再次下降，明确TTP诊断后于10月10日予以利妥昔单抗治疗，PLT仍持续下降且出现病情加重，于10月16～18日连续3 d予以卡拉西珠单抗治疗，PLT迅速回升至正常水平；**B** 乳酸脱氢酶（LDH），启动初始治疗后LDH有所下降，但随着病情再次加重，LDH明显升高，待患者病情控制，血小板水平明显回升，LDH也随之下降至正常水平；**C** 间接胆红素（IBIL），随着患者病情逐渐缓解，微血管内溶血得到控制，IBIL水平明显下降

## 讨论及文献复习

TTP临床进程常呈暴发性，多器官微血管血栓形成可迅速导致不可逆的终末器官损伤，病死率极高[2]。因此，早期识别与紧急干预是决定预后的关键[1],[7]–[8]。鉴于检测ADAMTS13活性需时较长，临床实践中高度依赖基于MAHA与血小板减少症“二联征”的推定诊断[9]。TPE是急性起病TTP治疗的基石，可清除自身抗体、补充有功能的ADAMTS13酶及阻断新的微血栓形成[10]。任何治疗时机的延误都将显著升高患者病死率，加剧器官损伤，甚至导致不可逆的功能障碍[11]–[12]。针对首次急性发作，2020年国际血栓与止血学会（ISTH）指南建议，将TPE联合皮质类固醇作为一线方案；有条件情况下，推荐联合利妥昔单抗与卡拉西珠单抗[7]。本中心的一项回顾性分析也表明，尽早启动TPE可降低病死率，使PLT快速恢复正常，同时前线加用利妥昔单抗可以明显改善患者预后[13]。

难治性TTP定义：去除诱因后，经5次血浆置换联合糖皮质激素治疗无临床反应（PLT持续低于50×10^9^/L，并且LDH持续大于1.5倍正常值上限）[14]–[15]。在诊断为难治性TTP前，重新评估临床情况以鉴别其他可能导致血小板减少和MAHA的原因非常有必要[9]。利妥昔单抗能提高ADAMTS13活性，缩短PLT恢复和TPE持续时间，在30 d内诱导血小板持续反应[16]–[17]。需注意的是，利妥昔单抗的临床疗效通常于首次给药后1～2周显现；在此窗口期内，患者仍面临病情反复与恶化乃至死亡的风险[18]。卡拉西珠单抗是一种靶向超大分子量vWF多聚体A1结构域的纳米抗体，能够阻止与血小板GPⅠb-Ⅸ-Ⅴ受体的相互作用，防止微血栓形成，预防微循环阻塞及缺血性器官损伤[2]。临床研究证实，iTTP患者在标准治疗（TPE、皮质类固醇及利妥昔单抗）基础上加用卡拉西珠单抗可获得更快、更持久的PLT恢复，这一优势在早期即联用利妥昔单抗的情况下依然存在[16],[19]。本例患者在第1次使用利妥昔单抗后，PLT仍持续下降，且出现明显神志障碍，病情加重，联合使用卡拉西珠单抗后PLT能迅速回升至正常水平。卡拉西珠单抗已于2018年9月和2019年2月先后获得欧洲药品管理局及美国食品药品监督管理局批准，用于iTTP治疗；2025年11月，该药正式获得中国国家药品监督管理局批准上市，用于治疗成人及12岁以上青少年iTTP患者，为国内该类罕见急危重症的救治提供了新的靶向选择。TITAN研究[6]及HERCULES研究[5]证实，无论是一线应用还是作为耐药/复发患者的挽救方案，卡拉西珠单抗均展现出显著效果。

在本例患者的治疗过程中，受限于血浆资源的短缺，初期血浆置换量（1 500 ml/d）未达指南推荐的40～60 ml/kg标准。然而，临床上通过联合应用5％白蛋白补足了置换缺口，以维持循环容量并确保抗体的持续清除[20]。虽然白蛋白有助于抗体移除且能降低输血相关过敏反应风险，但由于其缺乏ADAMTS13补给，微循环栓塞难以快速控制。正是在这种置换强度受限的背景下，卡拉西珠单抗通过即刻截断vWF-血小板相互作用，为后续免疫抑制治疗起效赢得了核心时间窗。患者经5 d TPE治疗后，PLT持续低于50×10^9^/L，并且LDH持续大于1.5倍正常值上限并出现新的神经系统症状，符合《血栓性血小板减少性紫癜诊断与治疗中国指南（2022年版）》[4]中对于难治性TTP的定义。加用卡拉西珠单抗后快速起效印证了在TPE等传统手段受限时，靶向vWF的病因学治疗的重要价值。本例患者实际接受了共4剂卡拉西珠单抗的短程冲击治疗，疗程显著短于标准临床试验设计及指南推荐的“每日1次直至最后1次血浆置换后30 d”[4]–[5]，但由于常规治疗（如利妥昔单抗）通常需1～2周起效，卡拉西珠单抗早期介入能有效阻断微血管血栓形成，显著缩短PLT恢复时间，降低病死率和难治率[21]–[22]。2024年的一项队列研究证实了基于ADAMTS13活性指导的减量方案，其中位给药仅6剂即可达到与标准疗程相当的临床转归[23]。本例患者在首剂给药24 h后PLT即显著回升，提示早期介入成功遏制了病理级联反应；此外，德国及西班牙的真实世界研究亦观察到超过半数患者疗程短于30 d，通过动态监测ADAMTS13活性指导停药是安全的[24]–[25]。因此，在药物可及性受限且病情危重的极端环境下，这种个体化策略具有一定的现实参考意义，但仍建议在有条件情况下严格遵循标准疗程。同时，在本例救治过程中，患者加用卡拉西珠单抗后，GCS评分迅速由9分恢复至14分，神经系统症状明显改善。这与文献报道的卡拉西珠单抗对iTTP神经系统受累的挽救疗效高度吻合[22],[26]。这种快速缓解得益于该药通过阻断脑部微血管内的血栓形成，有效缓解组织缺血[5]。真实世界数据显示，早期应用卡拉西珠单抗能显著缩短患者在重症监护室的停留时间，并大幅降低远期神经心理后遗症的发生率[21]。根据现有研究和前文所述，卡拉西珠单抗本身并不直接升高ADAMTS13活性或降低其抑制性抗体。其核心作用在于快速阻断vWF介导的血小板聚集和微血栓形成，从而遏制疾病的进展，为免疫抑制治疗和TPE清除自身抗体、恢复内源性ADAMTS13活性赢得了关键时间[9],[21]–[22]。本例患者ADAMTS13活性的恢复可能主要归因于免疫抑制治疗+TPE的持续作用，而卡拉西珠单抗通过打破“微血栓形成-器官缺血损伤”的恶性循环，为上述治疗创造了更有利的体内环境。

尽管卡拉西珠单抗的疗效已经得到证实，但究竟是将其作为治疗iTTP的一线药物还是仅用于难治性病例，也存在争议。目前已有病例和研究在评估卡拉西珠单抗联合糖皮质激素和利妥昔单抗而不进行TPE的治疗方案，这在血浆短缺的情况下，或是对于难以获取静脉通路/对血浆有严重免疫反应史而无法或不愿接受血浆置换的TTP患者可能具有重要意义[27]–[28]。出血是卡拉西珠单抗的主要不良反应，治疗过程中需密切关注新发出血情况[2]。

综上所述，本文展示了1例急性起病、对初始TPE及糖皮质激素治疗反应不佳的难治性iTTP。对于此类患者，早期识别、基于PLASMIC高危评分快速启动治疗对降低死亡率及改善远期预后至关重要。在疾病进展为明确的难治性阶段后，及时引入卡拉西珠单抗治疗，通过与利妥昔单抗及TPE联合，有效阻止了终末器官损伤，提升疗效，最终促使患者病情得以稳定。

## Supplementary Material


