# Supplementary figures and images for: Caplacizumab combined with therapeutic plasma exchange, glucocorticoids, and rituximab for refractory thrombotic thrombocytopenic purpura: a case report and literature review
Source: Zhonghua Xue Ye Xue Za Zhi. 2026 Mar;47(3):280–4. [Article in Chinese] doi: 10.3760/cma.j.cn121090-20251126-00553 (PMC13103595; doi:10.3760/cma.j.cn121090-20251126-00553)

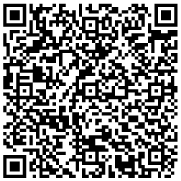

Supplement: Supplementary file 1 [file cjh-47-03-280-g002.tif]
